# Effect of Processing and Sintering on Controlled Release Wax Matrix Tablets of Ketorolac Tromethamine

**DOI:** 10.4103/0250-474X.58188

**Published:** 2009

**Authors:** Monica R. P. Rao, Anuradha A. Ranpise, K. C. Thanki, S. G. Borate, G. N. Parikh

**Affiliations:** AISSMS College of Pharmacy, Kennedy Road, Near RTO, Pune-411 001, India

**Keywords:** Controlled release, scanning electron microscopy (SEM), sintering, wax

## Abstract

The objective of present study was to evaluate the effect of processing methods and sintering condition on matrix formation and subsequent drug release from wax matrix tablets for controlled release. Ketorolac tromethamine and compritol were processed with appropriate diluent using either dry blending, spray drying, partial melt granulation or melt granulation.The tablets were then sintered at 80°. The sintered tablets were characterized by their physical parameters and *in vitro* dissolution tests. The micro-morphology and wettability of the tablets was also investigated. It was evident that different processing methods for identical formulation significant impact the release profile of drug. Sintering further retarded drug release and its effect was related to the manufacturing processes. Scanning electron microscopy showed that heat treatment redistributed the wax and formed a film-like structure covering drug and excipient particle. The contact angle of tablets made by dry blending, spray drying and partial melt granulation methods increased after sintering, while that of tablets made by melt granulation remained constant. Drug release from the wax tablets with or without heat treatment was best described by the Higuchi equation. Different processing methods produced different matrix structures that resulted in different drug release rates. Sintering retarded drug release mainly by decreasing the porosity of the matrix. Contact angle measurement and SEM analysis indicated that heat treatment caused the wax to melt, redistribute, coat the drug and diluents and form a network structure. Differential scanning calorimetry studies ruled out the occurrence of solid solution of the drug during sintering condition.

Controlled release drug delivery has become the norm in dosage form design and intensive research has been undertaken in achieving better drug product effectiveness reliability and safety. Wax has been used as matrix in pharmaceutical controlled release dosage form since decades[1]. Hydrophobic polymers (waxes) provide several advantages that include good stability at varying pH and moisture levels and effective retardation of highly water-soluble drug from the matrix[2]. Sintering is defined as the bonding of adjacent particle surfaces in a mass of powder or in a compact by application of heat[3]. This concept in pharmaceutical science is relatively recent, but research interests relating to this process have been growing. According to studies carried out, controlled release polymeric systems of rifampacin were prepared by mixing the drug and ethylene vinyl acetate copolymer, which were then compressed at room temperature. These matrices were exposed at 60°, 70° and 80° for 1.5, 3 and 4.5 h for sintering. The sintering time markedly affected the drug release properties of the ethylene vinyl acetate copolymer matrices. The percent release decreased as the sintering temperature was increased, for all formulation[4,5]. The release followed a diffusive mechanism, with first-order release kinetics. A similar effect was seen when rifampicin was replaced with theophylline[6] and when ethylene vinyl acetate copolymer was replaced with Eudragit RL100 matrices[7]. In another approach, thermal treatment of tablets containing Eudragit RS or RL in their structure above the glass transition temperature of the respected polymer was found to decrease drug release[8,9].

Ketorolac tromethamine is a non-steroidal antiinflammatory drug (NSAID), usually given in the treatment of rheumatoid arthritis, osteoarthritis and ankylosing spondylitis. The dose of conventional tablet is 10 mg three times a day. Due to high water solubility and short plasma half-life development of oral controlled release formulation of this drug is highly desirable[10,11]. The current study aimed to investigate the effect of processing methods and sintering condition on matrix formation and subsequent drug release from wax matrix tablets for controlled release.

## MATERIALS AND METHODS

Gift sample of ketorolac tromethamine USP was provided by Ranbaxy Lab Ltd, Goa, India. Compritol was provided by Gattefosse, France while lactose and talc by Loba Chemie Ltd. Mumbai, India. All chemicals were used as received.

### Dry blending (DB):

Ketorolac tromethamine was mixed with excipients (except lubricant) by geometric mixing in a polyethylene bag for 10 min. Then lubricant was added and mixed for an additional 5 min and the final blend was directly compressed using 6 mm punches on a rotary tablet compression machine (Mini Press II MT, Rimek). Hardness of tablets was kept to 5-6 kg/cm^2^.

### Spray dried blend (SD):

Ketorolac tromethamine was dissolved in methanol and mixed with a solution of Compritol 888 ATO in chloroform. This mixture was then spray-dried using spray dryer (LU-222, Labultima, Japan). The inlet and outlet drying air temperature was set to 60° and 40°, respectively. Feed pump rate of the solution was 5 ml/min. The resulting dried powder was mixed with excipients by geometric mixing. Finally the blend was lubricated and compressed using rotary tablet compression machine fitted with 6 mm punches. Hardness of tablets was kept to 5-6 kg/cm^2^.

### Partial melt granulation (PMG):

Compritol 888 ATO was melted at 75-80° in a porcelain evaporating dish on a water bath. Ketorolac tromethamine was gradually added to the molten wax with continuous stirring. The hot mass was cooled to 40-45° and then passed through sieve No. 18. The formed granules were mixed with excipients by geometric mixing and compressed using a rotary tablet machine. Hardness of tablets was kept to 5-6 kg/cm^2^.

### Melt granulation (MG):

Compritol 888 ATO was melted at 75-80° in a porcelain evaporating dish on a water bath. The drug and diluent mixture was gradually added to the molten wax with continuous stirring. The hot mass was cooled to 40-45° and then passed through 18 # sieve. The formed granules were lubricated and compressed using rotary tablet machine. Hardness of tablets was kept to 5-6 kg/cm^2^.

### Sintering of matrix tablets:

The tablets were then subjected to thermal treatment by placing on aluminium foil and subjected to sintering[3,8,9] at 80° for 1, 2 and 3 h in hot air oven (Labhosp, Mumbai, India).

### *In vitro* release studies:

Drug release was evaluated by conventional *in vitro* dissolution testing. The dissolution test for wax matrix tablet was performed in triplicate using dissolution test apparatus II (DA 6D Veego) at 100 rpm in 900 ml distilled water at 37°. Aliquots of 5 ml were periodically withdrawn and the sample volume replaced with an equal volume of fresh dissolution medium. The samples were filtered through Whatman filter paper and diluted ten times with distilled water. The diluted samples were analyzed at 322 nm by UV Spectrophotometer (Jasco, V-530, Japan). Cumulative percentage drug release was calculated using PCP Disso v2.08 Software (Poona College of Pharmacy, Pune, India).

### Wettability[12]:

The contact angle between purified water and tablet surfaces was determined by placing a 10 μl of water on surface of tablet using micropipette. Photographs of the drop in contact with water were taken. The drop was photographed after 10 s. It was carefully superimposed on tracing paper and the contact angle was measured. Amaranth red was added to the water to ensure proper visibility of the drop.

### Scanning electron microscopy (SEM):

SEM photomicrographs were taken by scanning electron microscope for studying surface morphology of matrix tablet before and after sintering. Each sample was mounted on to aluminum stub using double-sided adhesive tape and then coated with gold palladium alloy using Jeol/EO fine coat sputter. The surfaces of tablet were coated with platinum under an argon atmosphere. The samples were then examined with 200X and 1000X magnification using scanning electron microscope (Jeol JSM-6360A).

### Fourier transform infrared spectroscopy (FTIR):

FTIR spectra (ranging 400-4000 cm^−1^) of ketorolac tromethamine, Compritol 888 ATO, a physical mixture of ketorolac tromethamine and Compritol 888 ATO before and after heat treatment were investigated using (460 plus, Jasco) using the KBr disk method.

### Differential Scanning Calorimetric studies (DSC):

The DSC thermograms of ketorolac tromethamine, compritol 888 ATO, a physical mixture of ketorolac tromethamine and Compritol 888 ATO before and after sintering were recorded using Differential scanning calorimeter (DSC 823 Mettler Toledo, Japan). Approximately 2 to 5 mg of each sample was heated in a closed pierced aluminum pan from 30° to 300° at a heating rate of 10 °/min under a stream of nitrogen at a flow rate of 50 ml/min.

## RESULT AND DISCUSSION

**Fig. 1:**
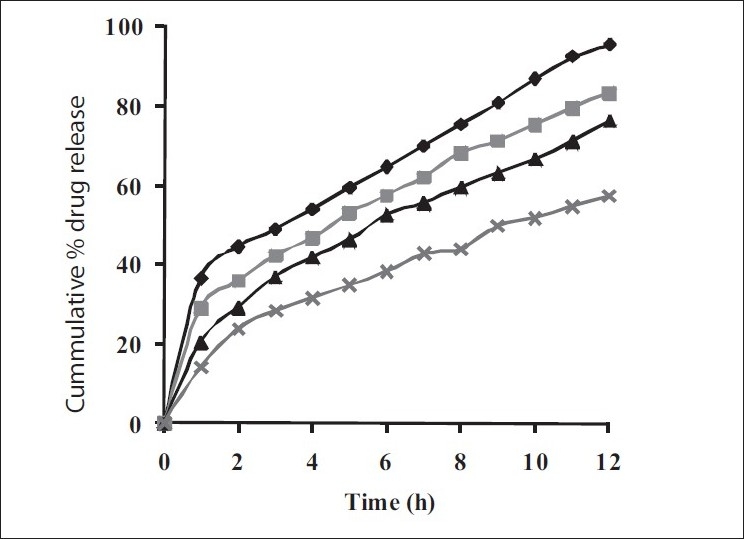
Comparative *in vitro* drug release evaluation of tablets prepared by different methods Comparative evaluation of *in vitro* drug release from tablets prepared from (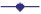) dry blending, (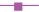) spray drying, (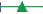) partial melt granulation and (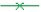) melt granulation.

**Fig. 2:**
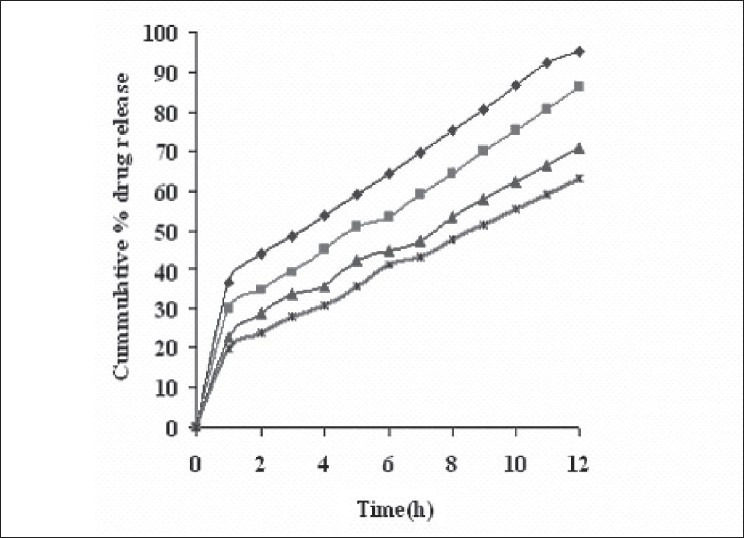
*In vitro* drug release from sintered matrix tablets prepared by dry blend method *In vitro* drug release from sintered matrix tablets prepared using direct compression method; (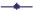) unsintered, (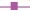) sintered for 1 h, (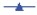) sintered for 2 h and (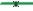) sintered for 3 h

**Fig. 3:**
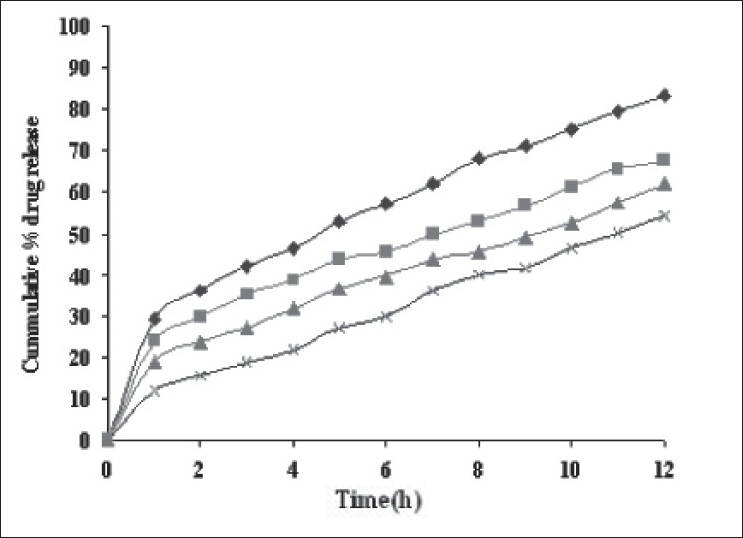
*In vitro* drug release from sintered matrix tablet prepared by spray dried method *In vitro* drug release from sintered matrix tablet prepared using spray dried method; (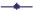) unsintered, (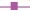) sintered for 1 h, (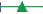) sintered for 2 h and (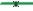) sintered for 3 h.

**Fig. 4:**
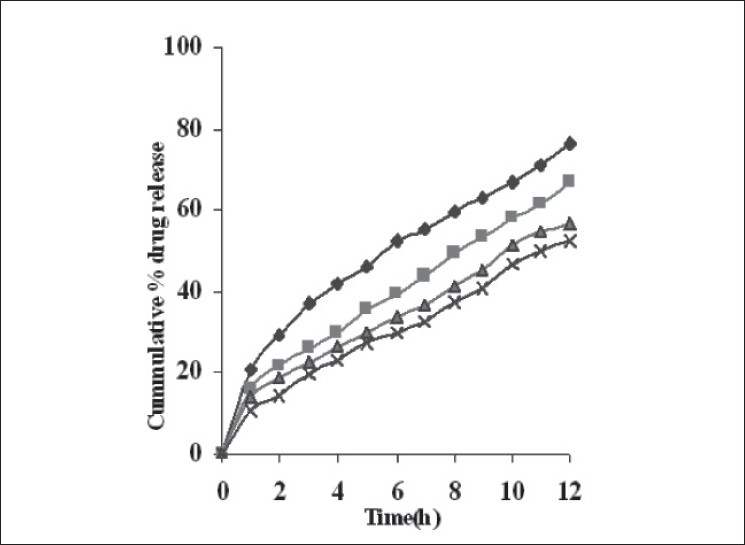
*In vitro* drug release from sintered matrix tablet prepared by partial melt granulation method *In vitro* drug release from sintered matrix tablet prepared using partial melt granulation method; (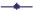) unsintered, (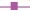) sintered for 1 h, (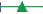) sintered for 2 h and (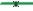) sintered for 3 h.

**Fig. 5:**
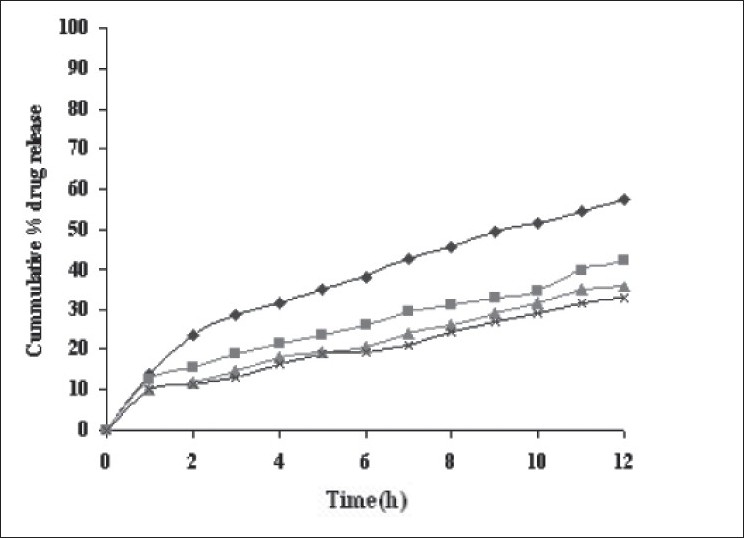
*In vitro* drug release from sintered matrix tablet prepared by melt granulation method *In vitro* drug release from sintered matrix tablet prepared using melt granulation method; (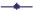) unsintered, (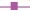) sintered for 1 h, (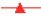) sintered for 2 h and (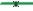) sintered for 3 h.

Dissolution profiles from all four formulations were compared. Although the same ingredients were present, each manufacturing process resulted in different dissolution profile (fig. 1). Tablets prepared by PMG and MG showed 76.25% and 57.37% drug release in 12 h, respectively, which indicates slower drug release than those prepared by DB and SD i.e. about 95.35% and 83.33% in 12 h. This could be due to the formation of different matrix structures from the different methods. The faster drug release from tablets prepared by DB and SD also indicates that, heat generated during compression was not high enough to melt the wax and form internal lattice structure similar to that for MG and PMG. All processing methods before and after sintering showed the Higuchi diffusion controlled matrix model for drug release. The *in vitro* dissolution study of sintered and unsintered matrix tablets of DB, SD, PMG and MG are shown in figs. 2–5. Drug release was slower after sintering for all four processing methods. In direct compression/dry blend (DB) method unsintered tablet showed 95.53% drug release in 12 h but after sintering at 80° for 1, 2 and 3 h drug release is decreased to 86.14, 71.03 and 63.25 %, respectively. In spray dried method (SD) unsintered tablet showed 83.33% drug release in 12 h but after sintering at 80° for 1, 2 and 3 h drug release was decreased to 67.63, 62.05 and 54.38%, respectively. In partial melt granulation (PMG) method unsintered tablet showed 76.25% drug release in 12 h but after sintering at 80° for 1, 2 and 3 h drug release was decreased to 66.84, 56.84 and 52.41%, respectively. In melt granulation (MG) method unsintered tablet show 57.37% drug release in 12h but after sintering at 80° for 1, 2 and 3 h drug release was decreased to 42.62, 35.71 and 32.56%, respectively. It was observed that sintering temperature and time markedly affected the drug release property of Compritol® ATO 888 matrices. Table 1 indicates kinetic treatment of drug release from controlled release tablets prepared by sintering of four different granulation methods. The same drug release mechanism for DC, SD, PMG and MG method with or without sintering means that the rate-controlling agent, wax, is responsible for the release mechanism. Processing methods and heat treatment may change the structure of wax matrix and influence the drug release but they do not change the drug release mechanism in these formulations.

**TABLE 1:** KINETIC TREATMENT OF DRUG RELEASE FROM CONTROLLED RELEASE TABLETS PREPARED BY SINTERING OF FOUR DIFFERENT GRANULATION METHODS

Formulation	R	n	k	Best Fit Model
Unsintered (DB)	0.9934	0.4613	27.58	Matrix model
Sintered (1h)	0.9928	0.4879	24.13	Matrix model
Sintered (2h)	0.9891	0.4508	25.13	Matrix model
Sintered (3h)	0.9886	0.4989	19.15	Matrix model
Unsintered (SD)	0.9979	0.4540	26.89	Matrix model
Sintered (1h)	0.9932	0.4739	25.49	Matrix model
Sintered (2h)	0.9890	0.5031	22.34	Matrix model
Sintered (3h)	0.9844	0.514	16.76	Matrix model
Unsintered (PMG)	0.9983	0.5227	19.92	Matrix model
Sintered (1h)	0.9893	0.4932	24.31	Matrix model
Sintered (2h)	0.9832	0.5316	18.81	Matrix model
Sintered (3h)	0.9869	0.5349	20.93	Matrix model
Unsintered (MG)	0.9973	0.5378	14.99	Matrix model
Sintered (1h)	0.9827	0.5139	14.96	Matrix model
Sintered (2h)	0.9963	0.4707	15.55	Matrix model
Sintered (3h)	0.9940	0.3933	18.01	Matrix model

**Fig. 6:**
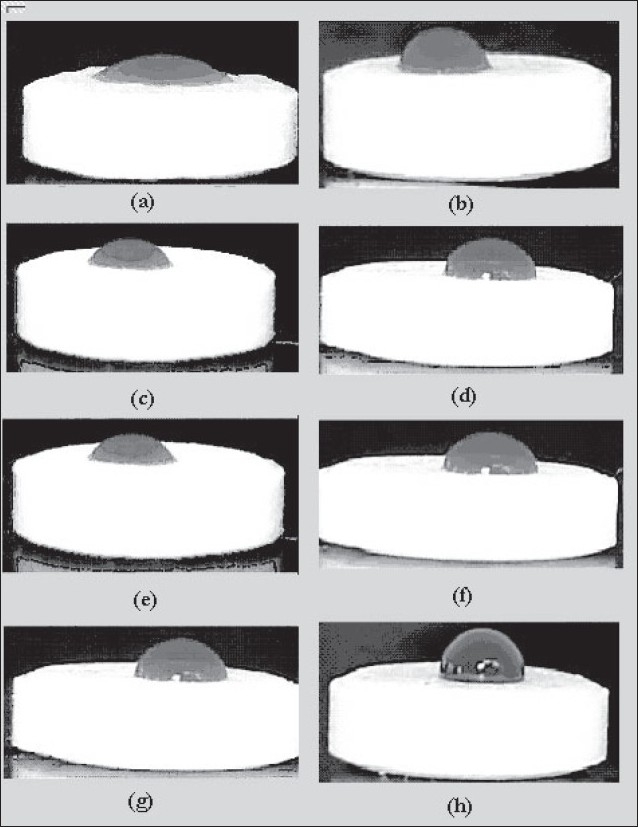
Photoimages showing contact angle of water with tablet surfaces made by different methods before and after sintering Photoimages showing contact of water with tablet surfaces made from different methods before and after sintering. (a) Unsintered dry blending, (b) Sintered dry blending. (c) Unsintered spray drying, (d) Sintered spray drying, (e) Unsintered partial melt granulation, (f) Sintered partial melt granulation, (g) Unsintered melt granulation, (h) Sintered melt granulation.

The effect of sintering on the wettability of the tablet surfaces was established by taking the photographs of the tablet surfaces on which drop of colored water was placed. Contact angle is indicative of the wettability of the tablet surface. Contact angle will be larger than 90° indicates greater hydrophobicity. It is an important physical property that has a far-reaching impact on the release of the drug especially from a hydrophobic wax matrix. The contact angle of DB, SD, PMG and MG tablet is 23°, 30°, 36° and 102°, respectively. As processing method changes the structure of matrix tablet and distribution of waxes on surfaces are different, hence leading to change in wettability of surface of tablets. The surfaces of tablets were dependent on the granulation method. The surface of the tablets made from dry blending consisted of the heterogeneous mixture of the excipients. The concentration of hydrophobic wax at tablet surface was lower in case of dry blending method than that in the melt granulation technique. Therefore, these tablets are less hydrophobic and have a smaller contact angle. On the other hand, tablets made from melt granulation contained granule that may act like drug and diluents coated with melted wax. Therefore, the granule surfaces may consist of a layer of melted wax. As a result, tablets show a higher contact angle. After sintering the hydrophobic wax is more uniformly dispersed through the compact and this leads to a decrease in the wettability of the tablet surface (fig. 6). This is evident by the higher contact angle which further contributes to the retardation of drug release from matrix tablet. Sintered tablets made from DB, SD, PMG and MG granulation methods after 3 h sintering show contact angle value 78°, 89°, 95° and 110°, respectively which indicated that the hydrophobic properties of surfaces after sintering were increased. This correlated well with the SEM micrographs. The lower wettability is responsible for retardation of drug release due to less penetration of dissolution medium in matrix tablet.

**Fig. 7:**
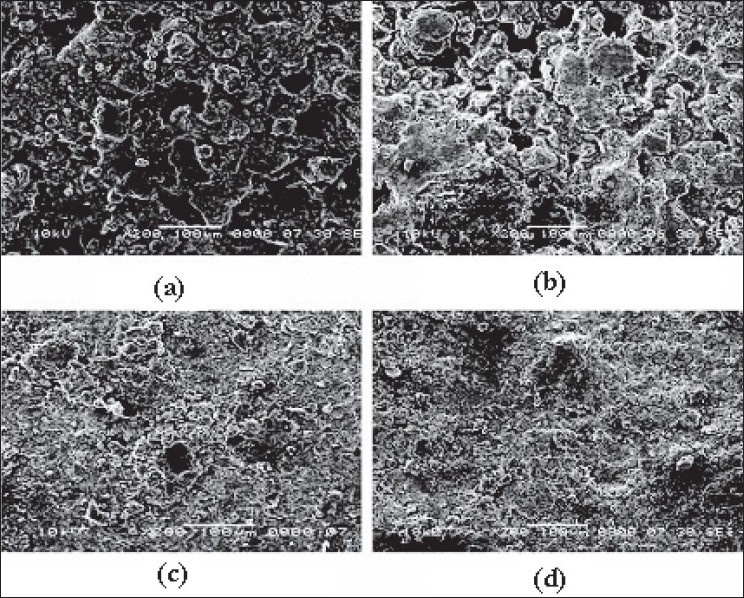
SEM of tablet surfaces made by different methods SEM of tablet surfaces made from different methods (200X). (a) dry blend tablet, (b) spray dried blend tablet, (c) partial melt granulation tablet, (d) melt granulation tablet

**Fig. 8:**
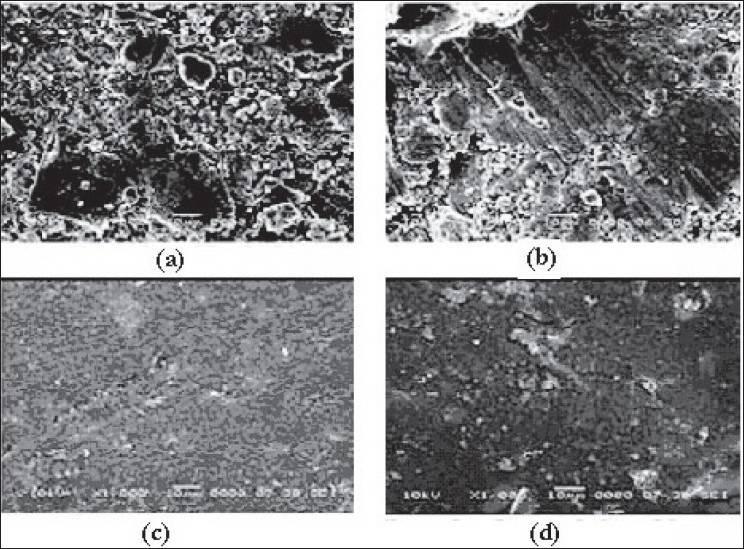
SEM of sintered matrix tablet surfaces (80° at 3h) made from different methods SEM of sintered matrix tablet surfaces (80° at 3h) made from different methods (1000X). (a) Sintered dry blend tablet, (b) Sintered spray dried blend tablet, (c) Sintered partial melt granulation tablet, (d) Sintered melt granulation tablet

Fig. 7 shows the surfaces of tablets made by DB, SD, PMG and MG methods. The surface of the tablets made from DB and SD showed heterogeneous mixture of excipients and consist more void spaces, indicating porous structure. On the other hand, tablets made by melt granulation seemed to be coated with wax had more uniform distribution of wax and the particles. Therefore, the tablet surface consisting less number of void spaces in matrix structures indicating less porosity. During sintering, the wax melts and gets redistributed. SEM micrographs (fig. 8) of the surfaces after sintering show that a thin, film-like structure covers the entire surface. There appears to be complete absence of any void spaces in these tablets indicating that heat treatment causes the wax to melt, redistribute and coat drug and excipient particles, thus creating new surfaces with lower wettability. This justifies the retardation of drug release from matrix tablets.

**Fig. 9:**
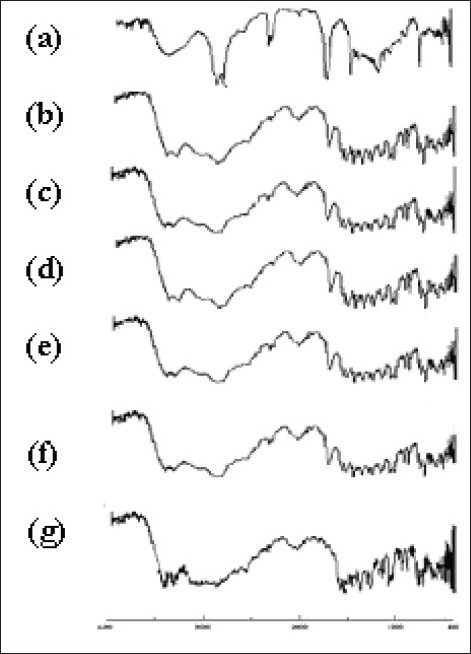
FTIR of ketorolac tromethamine, Compritol® 888 ATO, physical mixture (control) and sintered tablets made from different methods FTIR of (a) Compritol 888 ATO, (b) physical mixture (control), (c) sintered dry blend, (d) sintered spray dried blend, (e) sintered partial melt granulation, (f) sintered melt granulation, (g) ketorolac tromethamine.

To investigate the interaction during sintering, the FTIR spectra of ketorolac tromethamine, Compritol® 888 ATO, a physical mixture of ketorolac tromethamine and Compritol® 888 ATO before and after sintering were recorded (fig. 9). In the spectrum of ketorolac tromethamine major peaks 3350 cm^−1^ [NH stretch]; 1725 cm^−1^ [C=O stretch (acid)] 1167 cm^−1^ [C=O stretch (Diaryl ketone)] and 3450 cm^−1^ [OH (acid)] were seen in subsequent spectra. Comparison of the spectra of physical mixtures and heat-treated samples of ketorolac tromethamine with Compritol® 888 ATO showed no difference in the position of the absorption bands. The spectra can be simply regarded as the superposition of those of ketorolac tromethamine and Compritol® 888 ATO. This observation ruled out the possibility of chemical interaction and complex formation between these two components by sintering.

**Fig. 10:**
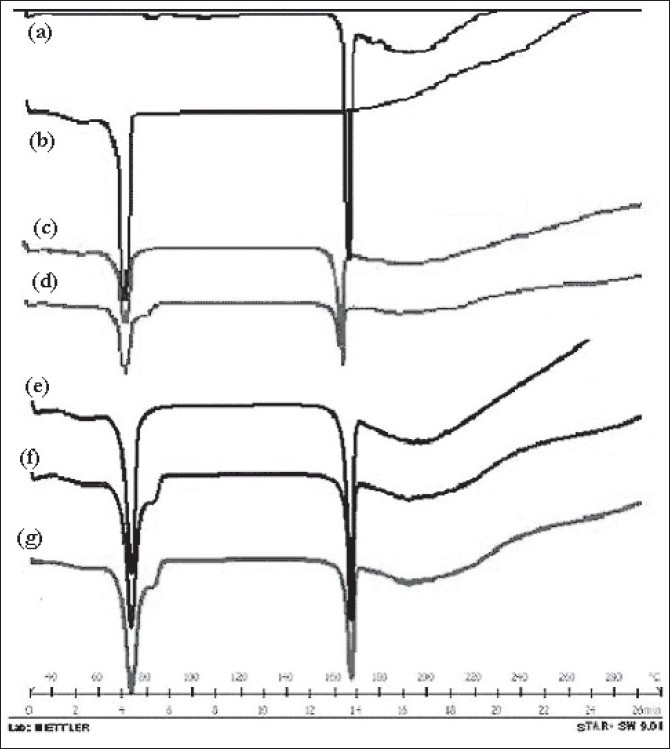
DSC thermograms of the pure drug and sintered tablets. DSC thermograms of the pure drug and sintered tablets. (a) ketorolac tromethamine, (b) Compritol 888 ATO, (c) physical mixture (control),(d) sintered dry blend, (e) sintered spray dried blend, (f) sintered partial melt granulation, (g) sintered melt granulation.

DSC spectra of ketorolac tromethamine, Compritol® 888 ATO, a physical mixture of ketorolac tromethamine and Compritol® 888 ATO before and after sintering are shown in fig. 10. DSC was used to examine thermal behavior of pure drug and formulation. The thermogram of ketorolac tromethamine shows a very sharp endothermic peak at 171° and Compritol 888 ATO shows sharp endothermic peak at approximately 72°. Comparison of the spectra of physical mixture (control) and sintered sample from all four methods shows that no change has occurred after sintering of sample. Therefore there is no evidence of drug interaction or complexation during manufacturing process and on sintering. DB, WG, PMG and MG of the same ingredients resulted in different matrix structures and different dissolution profiles. MG and PMG gave slower drug release compared to that of DB and WG. Sintering is defined as bonding of adjacent particle surfaces in a mass of compact by application of heat. Our study showed that drug release prolongation after sintering can be attributed to the melting and redistribution of the wax in the tablet matrix structure. Drug release from all tablets showed Higuchi diffusion controlled matrix release. Sintering of the tablets might have resulted in melting and redistribution of the wax throughout the matrix and a possible change in nature of the pores within the matrix. DSC and FT-IR studies ruled out the occurrence of solid solution and drug–wax interaction due to heat-treatment.
